# Deeskalation der adjuvanten Radiotherapie nach transoraler Resektion HPV-positiver Oropharynxkarzinome: Ergebnisse der E3311-Studie

**DOI:** 10.1007/s00066-022-01907-4

**Published:** 2022-02-08

**Authors:** Alexander Rühle, Nils H. Nicolay

**Affiliations:** 1 Klinik für Strahlenheilkunde, Universitätsklinikum Freiburg, Robert-Koch-Str. 3, Freiburg, Deutschland; 2 Deutsches Konsortium für Translationale Krebsforschung (DKTK), Partnerstandort Freiburg, Deutsches Krebsforschungszentrum (dkfz), Heidelberg, Deutschland

## Hintergrund und Ziel der Studie

Patienten mit HPV(Humanen Papillomavirus)-assoziierten Oropharynxkarzinomen weisen signifikant höhere Überlebensraten auf als Patienten mit anderen, HPV-negativen Plattenepithelkarzinomen im Kopf-Hals-Bereich. Aufgrund der guten Prognose einerseits und der andererseits häufig die Lebensqualität der Patienten beeinträchtigenden chronischen Toxizitäten prüfen derzeit zahlreiche Studien mögliche Deeskalationsstrategien mit dem Ziel, Nebenwirkungen der Radiotherapie (RT) zu reduzieren, ohne die guten onkologischen Ergebnisse dieser Patienten zu verschlechtern. In der hier diskutierten E3311-Studie wurden onkologische Effektivität und Toxizitäten einer transoralen Resektion (TOS) plus „neck dissection“ (ND) und einer risikoadaptierten dosisdeeskalierten postoperativen Radio(chemo)therapie (R[C]T) untersucht.

## Patienten und Methoden

Insgesamt 519 Patienten mit p16-positiven Oropharynxkarzinomen wurden in die randomisierte Phase-II-Studie eingeschlossen, von denen 495 eine TOS des Primärtumors mit ND erhielten. In Abhängigkeit der postoperativen Risikofaktoren wurden anschließend 445 Patienten den folgenden 4 Behandlungsarmen zugeteilt: Patienten mit niedrigem Rezidivrisiko (T1–2-Tumoren mit negativen Resektionsrändern [>3 mm] und limitierter lymphogener Metastasierung [N0–1, 7. AJCC-Edition] ohne Kapseldurchbruch) erhielten keine postoperative Therapie (*Arm A*). Patienten mit intermediärem Risiko (T1–2-Tumoren mit a. geringem Sicherheitsabstand [<3 mm] oder b. lediglich minimalem Kapseldurchbruch [≤1 mm] oder c. ≤ 4 Lymphknotenmetastasen oder d. perineuraler/lymphovaskulärer Invasion) wurden zwischen einer deeskalierten adjuvanten RT mit 50 Gy (*Arm B*) oder der Standardtherapie mit 60 Gy (*Arm C*) randomisiert. Dabei wurde bei der Randomisierung anhand des Raucherstatus (≤10 vs. >10 Packungsjahre) stratifiziert. Patienten mit histopathologischen Hochrisikofaktoren (positiver Resektionsrand, Kapseldurchbruch >1 mm und/oder ≥5 tumorbefallene Lymphknoten) wurden einer postoperativen RCT mit 66 Gy und konkomitanten wöchentlichen Cisplatingaben (40 mg/m^2^) zugeführt (*Arm D*).

## Ergebnisse

Nach einer medianen Nachbeobachtungszeit von 35,2 Monaten betrug das progressionsfreie Überleben (PFS) nach 2 Jahren bei den 359 evaluierbaren Patienten 96,9 % in Arm A, 94,9 % in Arm B, 96,0 % in Arm C und 90,7 % in Arm D. Die untere Grenze des 90 %-Konfidenzintervalls für das 2‑Jahres-PFS im Arm B lag bei 91,3 % und erfüllte damit den vorher festgelegten Endpunkt von ≥85 %. Die Rate an Grad 3–5-Toxizitäten war in Arm B signifikant geringer als in Arm C (14 % vs. 24 %, *p* = 0,030). Die mittels MDADI (MD Anderson Dysphagia Index) und FACT-HN („functional assessment of cancer therapy-head and neck“) quantifizierte Schluckfähigkeit war in Arm D signifikant schlechter als in den Armen B und C. Und gemessen am 6‑Monats-FACT-HN erholte sich die Schluckfähigkeit im deeskalierten Therapiearm B besser als in Arm C.

## Schlussfolgerung der Autoren

Bei Patienten mit p16-positiven Oropharynxkarzinomen resultierte eine TOS mit ND und risikoadaptierter postoperativer R(C)T in sehr guten onkologischen Ergebnissen mit signifikanter Verbesserung der Schluckfunktion durch eine Reduktion der adjuvanten Bestrahlungsdosis. Angesichts der hohen onkologischen Effektivität und der geringen Toxizitätsraten des deeskalierten Behandlungsarms für Patienten mit intermediärem Risiko soll dieser Arm nun in einer Phase-III-Studie gegen eine definitive Standard-RCT getestet werden.

## Kommentar

Die hier diskutierte ECOG-ACRIN-E3311-Studie markiert einen weiteren vielversprechenden Schritt der Therapiedeeskalation bei Patienten mit HPV-assoziierten Oropharynxkarzinomen. Während viele der bisher publizierten Studien, beispielsweise die drei randomisierten Studien zum Ersatz von Cisplatin durch Cetuximab (De-ESCALaTE HPV, RTOG 1016 und TROG 12.01), oder die beiden Dosisdeeskalationsstudien HN002 und 30-ROC Patienten mit definitiver RCT einschlossen [1], untersucht die vorliegende E3311-Studie die risikoadaptierte Dosisdeeskalation für die postoperative RT nach TOS. Aufgrund der hohen Patientenzahl sowie des multizentrischen Designs mit qualitätsgesicherter TOS ist die E3311-Studie neben der MC1273-Phase-II-Studie und der bisher nur als Konferenzbeitrag veröffentlichten Phase-III-Folgestudie MC1675 (Deeskalation der adjuvanten RT-Dosis auf 30 bzw. 36 Gy mit Einzeldosen von 1,5 bzw. 1,8 Gy zweimal täglich mit konkomitanter docetaxelbasierter Chemotherapie) eine der wichtigsten bisher vorliegenden Studien zur Therapiedeeskalation in der postoperativen Situation [2].

Beeindruckend in dieser multizentrischen TOS-Studie ist die hohe chirurgische Qualität: Insbesondere die Rate an R1-Resektionen (4,0 % in der finalen Qualitätsauswertung) ist deutlich geringer als in anderen Konkurrenzstudien oder Datenbanken. In den beiden prospektiven ORATOR-Studien, bei denen die robotergestützte TOS plus risikoadaptierte Adjuvanz mit einer definitiven RT verglichen wurde, lagen die Raten an inkompletten Resektionen bei 12 % (ORATOR) bzw. 8 % (ORATOR 2; [3, 4]). Eine aktuelle Auswertung der amerikanischen National Cancer Data Base (NCDB) zeigte sogar bei HPV-positiven pT1–2-Oropharynxkarzinomen positive Resektionsränder in fast einem Viertel der Fälle (23,4 %; [5]). Diese Daten werfen daher die Frage auf, inwieweit die Qualitätsvoraussetzungen der E3311-Studie auf die klinische Routineversorgung der Patienten zu übertragen sind. Die Häufigkeit von höhergradigen oropharyngealen Blutungen in der E3311-Studie (Grad 3–4: 5,9 %, Grad 3–5: 6,1 %) ist hingegen vergleichbar mit denen der beiden ORATOR-Studien: So betrug die Rate an höhergradigen (Grad 3–5) postoperativen oropharyngealen Blutungen in der initialen ORATOR-Studie 5,9 % und in der ORATOR-2-Studie 6,4 % [3, 4]. Anders jedoch als in den beiden ORATOR-Studien, bei denen es jeweils bei 3 % der Patienten zu letalen Blutungen kam, traten solche fatalen Ereignisse in der E3311-Studie nur sehr selten auf (0,2 %). Die im Amendment der E3311-Studie sehr empfohlene perioperative Gefäßligatur wird in diesem Zusammenhang vermutlich zu geringeren letalen Blutungsereignissen beigetragen haben. Die aktuell rekrutierende Phase-II/III-PATHOS-Studie (NCT02215265) zu TOS und risikoadaptierter postoperativer R(C)T wird mit der angestrebten Zahl von 1100 Patienten hoffentlich dazu beitragen, die Diskrepanzen hinsichtlich der chirurgischen Qualität zwischen der E3311-Studie und den ORATOR-Studien zu klären [6]. Auf jeden Fall bleibt angesichts der sehr guten Ergebnisse der E3311-Studie festzuhalten, dass Patienten mit Oropharynxkarzinomen, welche eine Resektion präferieren, von einer Behandlung in Zentren mit qualitätsgesicherter operativer Technik und hohen Fallzahlen profitieren [7].

Korrespondierend zu der in dieser Studie angewandten Unterscheidung zwischen limitiertem extranodalem Tumorwachstum (mikroskopischer Kapseldurchbruch ≤1 mm) und ausgeprägtem Kapseldurchbruch (makroskopischer Kapseldurchbruch >1 mm) konnten Bauer et al. zeigen, dass HPV-positive Oropharynxkarzinompatienten mit Kapseldurchbruch >1 mm ein schlechteres Gesamtüberleben aufwiesen als Patienten mit lediglich mikroskopischem Kapseldurchbruch [8]. Internationale Empfehlungen zur histopathologischen Befundung von Lymphknotenmetastasen im Kopf-Hals-Bereich haben 2 mm als Grenzwert zwischen mikroskopischem und makroskopischem Kapseldurchbruch angegeben [9]. Es bleibt jedoch abzuwarten, inwieweit dieser Parameter in Zukunft zur Therapieentscheidung herangezogen wird; andere Studien zur Therapiedeeskalation in der Adjuvanz wie beispielsweise die DELPHI-, MC1273-, MC1675- und PATHOS-Studie führen eine solche Unterscheidung bei Kapseldurchbruch nicht durch.

Eine zentrale prognostische Determinante für Patienten mit HPV-assoziierten Oropharynxkarzinomen ist der Raucherstatus. In der Post-hoc-Analyse der RTOG-0129-Studie zeigte sich nämlich, dass neben dem HPV-Status auch das Ausmaß des Nikotinabusus, ausgedrückt in Zigarettenpackungsjahren, einen wesentlichen Risikofaktor darstellt: So wiesen Ang und Kollegen darauf hin, dass Patienten mit >10 Packungsjahren und ausgeprägterem Nodalbefall (≥N2b, 7. AJCC-Edition) trotz HPV-positiver Histologie nicht mehr zur Niedrigrisikogruppe gezählt werden dürfen [10]. Die Diskrepanz zwischen der RTOG-0129-Studie, in der Patienten eine definitive RCT erhielten, und der vorliegenden E3311-Studie scheint darauf hinzudeuten, dass die Anzahl der Packungsjahre möglicherweise eine weniger starke prognostische Rolle bei einem primär chirurgischen Ansatz aufweist. Folgerichtig wurden daher auch in der MC1675-Studie (NCT02908477), einer chirurgischen Phase-III-Studie mit TOS und deeskalierter postoperativer Therapie, Patienten unabhängig vom Nikotinstatus eingeschlossen, wohingegen in der HN002-Studie, einer Phase-II-Studie zur Deeskalation der definitiven R(C)T, lediglich Patienten mit ≤10 Packungsjahren berücksichtigt wurden [11].

Erwähnenswert an dieser Studie ist die Tatsache, dass von den 445 auf die vier Behandlungsarme verteilten Patienten immerhin 138 Patienten, also fast ein Drittel (31 %), eine additive RCT benötigten und somit im Endeffekt trotz kleinen Primärtumors eine trimodale Therapie erhielten. In lediglich einem von 10 Fällen in Arm D war diese RCT wegen einer R1-Resektion erforderlich, wohingegen in der überwiegenden Mehrzahl (83,2 %) ein Lymphknotenkapseldurchbruch >1 mm vorlag und der Grund für eine postoperative RCT war. In Anbetracht der signifikant höheren Toxizitätsrate in Arm D, sowohl was die von Behandlern angegebenen CTCAE-Gradierung anbelangt (Grad-3–5-Toxizitäten: 14 % in Arm B vs. 24 % in Arm C vs. 60 % in Arm D) als auch die von Patienten berichtete Schluckfunktion (1-Jahres-MDADI: 79,1 in Arm B vs. 78,8 in Arm C vs. 73,3 in Arm D), muss dieser Aspekt in den aktuellen Deeskalationsdebatten weiterhin kritisch angemerkt werden. Verglichen damit erscheinen die onkologischen und toxizitätsbezogenen Endpunkte der HN002-Studie, einer randomisierten Phase-II-Studie zwischen einer dosisdeeskalierten definitiven RT mit 60 Gy und einer ebenfalls dosisdeeskalierten definitiven RCT mit 60 Gy bei HPV-positiven Oropharynxkarzinompatienten, vergleichbar bzw. was die Schluckfunktion anbelangt tendenziell überlegen: Das 2‑Jahres-PFS im dosisdeeskalierten RCT-Arm der HN002-Studie lag bei 90,5 % und der 1‑Jahres-MDADI bei 85,3 [11]. Auch die Daten der randomisierten ORATOR-Studie zeigten bei allen diskutierten Limitationen bereits eine bessere auf die Schluckfunktion bezogene Lebensqualität im RCT-Arm verglichen mit dem TOS-Arm (1-Jahres-MDADI 86,9 vs. 80,1, *p* = 0,042), obgleich dieser Unterschied nicht die Grenze zur klinischen Relevanz überschritt. Ob bei Hochrisikopatienten die konkomitante Chemotherapie in der Adjuvanz weggelassen werden kann, wird erst die PATHOS-Studie klären können. Die Rekrutierung dieser Studie wird jedoch voraussichtlich erst 2026 abgeschlossen sein. Bildgebende Verfahren, welche einen Lymphknotenkapseldurchbruch mit hoher Sensitivität und Spezifität voraussagen, wären eine vielversprechende Möglichkeit, solche Patienten in Zukunft tendenziell eher einer bimodalen (deeskalierten) Therapie (z. B. cisplatinbasierte definitive RCT mit 60 Gy analog HN002) zuzuführen. Zusammengefasst bleibt zum aktuellen Zeitpunkt das nicht unerhebliche Risiko von trimodalen Therapien im Falle eines TOS-basierten Deeskalationsansatzes, welcher im Vergleich zur auf 60 Gy deeskalierten bimodalen definitiven RCT eine Therapieeskalation darstellt. Auch die vorhandenen Daten zum „decision regret“ von Patienten mit Kopf-Hals-Karzinomen legen nahe, dass Patienten nach trimodalen Therapien signifikant häufiger die durchgeführte Therapieentscheidung bereuen als Patienten mit uni- oder bimodalen Therapien [12].

Obgleich den Autoren zum Gelingen dieser multizentrischen Phase-II-Studie und der hohen TOS-Qualität zu gratulieren ist, müssen die noch nicht ausreichend lange Nachbeobachtungszeit von im Median knapp 3 Jahren (HPV-positive Oropharynxkarzinompatienten zeigen nämlich häufiger als HPV-negative Kopf-Hals-Patienten im weiteren Verlauf noch lokoregionäre und vor allem distante Spätrezidive) sowie die Tatsache, dass lediglich der p16-Status als Surrogatparameter für den HPV-Status herangezogen wurde, als weitere Kritikpunkte benannt werden.

Zuletzt soll noch kurz auf den Umstand hingewiesen werden, dass bei ungefähr einem Viertel der Patienten mit intermediärem Rezidivrisiko (26 % in Arm B und 19 % in Arm C) auf die Bestrahlung der Primärtumorregion verzichtet wurde. Ein solches Vorgehen bei ausreichend weit im Gesunden resezierten HPV-positiven pT1–2-Oropharynxkarzinomen wurde beispielsweise auch in der AVOID-Studie, einer prospektiven Phase-II-Studie, und in einer monozentrischen retrospektiven Studie mit guten Ergebnissen beschrieben [13, 14]: Während in der AVOID-Studie lediglich bei einem der 60 eingeschlossenen Patienten ein Lokalrezidiv festgestellt wurde, trat in der retrospektiven Studie von Dhere et al. kein einziges lokoregionäres Rezidiv bei 59 Patienten auf.

## Fazit

Erstmals konnte in einer großen multizentrischen Studie gezeigt werden, dass eine primäre TOS mit anschließender risikoadaptierter deeskalierender R(C)T zu sehr guten onkologischen Ergebnissen bei Patienten mit HPV-assoziierten Oropharynxkarzinomen führt. Die Daten der Studie legen nahe, dass eine postoperative RT mit 50 statt mit 60 Gy bei intermediärem Rezidivrisiko zur lokoregionalen Tumorkontrolle ausreicht. Ob die hier geforderte chirurgische Qualität, mitverantwortlich für die herausragenden Ergebnisse der Studie, allerdings auf die breite „reale“ Welt flächendeckend übertragen werden kann, darf heute noch bezweifelt werden. Und die Notwendigkeit von trimodalen Therapien für beinahe ein Drittel der Studienkohorte zeigt auch, dass die optimale Deeskalationsstrategie bei den Patienten mit HPV-positiven Oropharynxkarzinomen noch nicht entschieden ist. Die von den Autoren geplante Phase-III-Studie zum Vergleich des auf 50 Gy deeskalierten TOS-Arms mit einer definitiven Standard-RCT ist daher sehr zu begrüßen.

Inwieweit Hochrisikopatienten nach einer Resektion auf die additive RCT verzichten dürfen und stattdessen nur mit einer alleinigen postoperativen RT behandelt werden können, wird hoffentlich in einigen Jahren durch die PATHOS-Studie beantwortet werden. Bis zum Vorliegen von positiven Phase-III-Daten sollte eine Deeskalation weiterhin nur im Rahmen von prospektiven Studien erfolgen.

*Alexander Rühle und Nils H. Nicolay, Freiburg*

## References

[1] Rühle A, Grosu A-L, Nicolay NH (2021). De-escalation strategies of (chemo)radiation for head-and-neck squamous cell cancers—HPV and beyond. Cancers.

[2] Ma DJ, Price KA, Moore EJ, Patel SH, Hinni ML, Garcia JJ (2019). Phase II evaluation of aggressive dose de-escalation for adjuvant chemoradiotherapy in human Papillomavirus-associated oropharynx squamous cell carcinoma. J Clin Oncol.

[3] Nichols AC, Theurer J, Prisman E, Read N, Berthelet E, Tran E (2019). Radiotherapy versus transoral robotic surgery and neck dissection for oropharyngeal squamous cell carcinoma (ORATOR): an open-label, phase 2, randomised trial. Lancet Oncol.

[4] Palma DA, Prisman E, Berthelet E, Tran E, Hamilton SN, Wu J (2021). A randomized trial of radiotherapy vs. trans-oral surgery for treatment de-escalation in HPV-associated oropharyngeal squamous cell carcinoma (ORATOR2). Int J Radiat Oncol Biol Phys.

[5] Zhan KY, Puram SV, Li MM, Silverman DA, Agrawal AA, Ozer E (2020). National treatment trends in human papillomavirus–positive oropharyngeal squamous cell carcinoma. Cancer.

[6] Owadally W, Hurt C, Timmins H, Parsons E, Townsend S, Patterson J (2015). PATHOS: a phase II/III trial of risk-stratified, reduced intensity adjuvant treatment in patients undergoing transoral surgery for human papillomavirus (HPV) positive oropharyngeal cancer. BMC Cancer.

[7] Ferris RL, Flamand Y, Holsinger FC, Weinstein GS, Quon H, Mehra R (2020). A novel surgeon credentialing and quality assurance process using transoral surgery for oropharyngeal cancer in ECOG-ACRIN Cancer Research Group Trial E3311. Oral Oncol.

[8] Bauer E, Mazul A, Chernock R, Rich J, Jackson RS, Paniello R (2020). Extranodal extension is a strong prognosticator in HPV-positive oropharyngeal squamous cell carcinoma. Laryngoscope.

[9] Bullock MJ, Beitler JJ, Carlson DL, Fonseca I, Hunt JL, Katabi N (2019). Data set for the reporting of nodal excisions and neck dissection specimens for head and neck tumors: explanations and recommendations of the guidelines from the international collaboration on cancer reporting. Arch Pathol Lab Med.

[10] Ang KK, Harris J, Wheeler R, Weber R, Rosenthal DI, Nguyen-Tân PF (2010). Human Papillomavirus and survival of patients with oropharyngeal cancer. N Engl J Med.

[11] Yom SS, Torres-Saavedra P, Caudell JJ, Waldron JN, Gillison ML, Xia P (2021). Reduced-dose radiation therapy for HPV-associated oropharyngeal carcinoma (NRG oncology HN002). J Clin Oncol.

[12] Windon MJ, D’Souza G, Faraji F, Troy T, Koch WM, Gourin CG (2019). Priorities, concerns, and regret among patients with head and neck cancer. Cancer.

[13] Swisher-McClure S, Lukens JN, Aggarwal C, Ahn P, Basu D, Bauml JM (2020). A phase 2 trial of alternative volumes of oropharyngeal irradiation for de-intensification (AVOID): omission of the resected primary tumor bed after transoral robotic surgery for human Papilloma virus-related squamous cell carcinoma of the Oropharynx. Int J Radiat Oncol Biol Phys.

[14] Dhere VR, Escott CE, Tian S, Switchenko JM, Bell JP, Stokes WA (2021). The omission of intentional primary site radiation following transoral robotic surgery in 59 patients: no local-regional failures. Head Neck.

